# BCL2 and BCL(X)L selective inhibitors decrease mitochondrial ATP production in breast cancer cells and are synthetically lethal when combined with 2-deoxy-D-glucose

**DOI:** 10.18632/oncotarget.25433

**Published:** 2018-05-25

**Authors:** Federico Lucantoni, Heiko Düssmann, Irene Llorente-Folch, Jochen H.M. Prehn

**Affiliations:** ^1^ Department of Physiology & Medical Physics, Royal College of Surgeons in Ireland, Dublin 2, Ireland; ^2^ Center for Systems Medicine, Royal College of Surgeons in Ireland, Dublin 2, Ireland

**Keywords:** breast cancer, BCL2 inhibitors, cell death, bioenergetics, OXPHOS

## Abstract

Cancer cells display differences regarding their engagement of glycolytic vs. mitochondrial oxidative phosphorylation (OXPHOS) pathway. Triple negative breast cancer, an aggressive form of breast cancer, is characterized by elevated glycolysis, while estrogen receptor positive breast cancer cells rely predominantly on OXPHOS. BCL2 proteins control the process of mitochondrial outer membrane permeabilization during apoptosis, but also regulate cellular bioenergetics. Because BCL2 proteins are overexpressed in breast cancer and targetable by selective antagonists, we here analysed the effect of BCL2 and BCL(X)L selective inhibitors, Venetoclax and WEHI-539, on mitochondrial bioenergetics and cell death. Employing single cell imaging using a FRET-based mitochondrial ATP sensor, we found that MCF7 breast cancer cells supplied with mitochondrial substrates reduced their mitochondrial ATP production when treated with Venetoclax or WEHI-539 at concentrations that *per se* did not induce cell death. Treatments with lower concentrations of both inhibitors also reduced the length of the mitochondrial network and the dynamics, as evaluated by quantitative confocal microscopy. We next tested the hypothesis that mitochondrial ATP production inhibition with BCL2 or BCL(X)L antagonists was synthetically lethal when combined with glycolysis inhibition. Treatment with 2-deoxy-D-glucose in combination with Venetoclax or WEHI-539 synergistically reduced the cellular bioenergetics of ER+ and TNBC breast cancer cells and abolished their clonogenic potential. Synthetic lethality was also observed when cultures were grown in 3D spheres. Our findings demonstrate that BCL2 antagonists exert potent effects on cancer metabolism independent of cell death-inducing effects, and demonstrate a synthetic lethality when these are applied in combination with glycolysis inhibitors.

## INTRODUCTION

One of the emerging hallmarks of cancers is the alteration of cellular metabolism, an essential property to maintain proliferation and growth [1]. Cancer cells often possess increased levels of aerobic glycolysis due to impairments in the OXPHOS pathway, a phenomenon termed the “Warburg effect”. In this pathway, pyruvate derived from glycolysis is converted to lactate by lactate dehydrogenase and subsequently secreted [2]. Even though the Warburg effect is a widespread phenomenon associated with cancer, OXPHOS function is still retained by most tumours. Within the same malignant population metabolic profiles may vary as cancer cells are able to adapt easily to changes in the surrounding environment due to their plasticity [3]. Estimated levels of glycolytic ATP production vary between 1-64% in different cancers [4]. Several studies showed that mitochondrial OXPHOS is still intact in cancer cells, but that active glycolysis suppresses OXPHOS [5–7]. In HeLa and estrogen receptor positive (ER+) MCF7 breast cancer cells it has been observed that OXPHOS contributes to 79 and 91% ATP generation respectively, with a reduction to 29 and 36% under hypoxia when glycolytic pathways are active [8]. On the other hand, triple negative breast cancer (TNBC), an aggressive subtype of breast cancer with poor prognosis and no targeted therapies, demonstrates higher glycolytic flux and lower OXPHOS function [9, 10].

Proteins of the BCL2 family control the process of mitochondrial outer membrane permeabilization (MOMP) during apoptosis [11]. However, BCL2 proteins also regulate the bioenergetics status of cells through their control of mitochondrial fusion and fission dynamics, and may act directly on the mitochondrial respiratory chain [12–16]. For example, BCL2 overexpression in human leukaemia cells increased oxygen consumption and mitochondrial respiration [15]. In neurons, a pool of the anti-apoptotic BCL(X)L protein has been found to localise in the inner mitochondrial membrane (IMM) and to interact with F_1_F_0_ ATP synthase, increasing its enzymatic activity and stabilizing mitochondrial membrane potential (ΔΨ_m_) [14, 17]. A truncated form of the anti-apoptotic MCL1 protein has been shown to localize to the mitochondrial matrix and to increase mitochondrial respiration [18]. Because anti-apoptotic BCL2 family proteins confer resistance of cancer cells to therapy, several selective inhibitors that target individual or multiple anti-apoptotic BCL2 proteins, have been developed [19]. The first BCL2 selective inhibitor Venetoclax (ABT199) has been approved for clinical use for the treatment of chronic lymphocytic leukemia with 17p deletion [20]. However, little information is currently available whether selective inhibition of BCL2 or other anti-apoptotic BCL-2 protein such as BCL(X)L also influences mitochondrial function and energetics in cancer cells [21], and whether these functions can be exploited therapeutically. We have previously observed that treatment of triple negative breast cancer (TNBC) cells with Venetoclax or the selective BCL(X)L inhibitor WEHI-539 synergistically increased responses to the cytotoxic agent cisplatin, but that responses could not be fully explained by an increased sensitivity of the cells to undergo MOMP [22]. In the present study, we therefore explored whether selective BCL2 or BCL(X)L inhibition impacted on mitochondrial bioenergetics using a combined high content analysis and single-cell Foerster resonance energy transfer (FRET)-based imaging approach, and investigated how these effects related to cell death induction by BCL2 and BCL(X)L inhibitors. Our data demonstrate that BCL2 and BCL(X)L inhibition profoundly impacts on mitochondrial ATP production, and that a combined treatment of BCL2 or BCL(X)L selective inhibitors and glycolysis inhibitors produced synthetic lethality in estrogen receptor positive (ER+) and TNBC breast cancer cells.

## RESULTS

### Low concentrations of BCL2 inhibitors alter mitochondrial activity without affecting cell death

**Figure 1:**
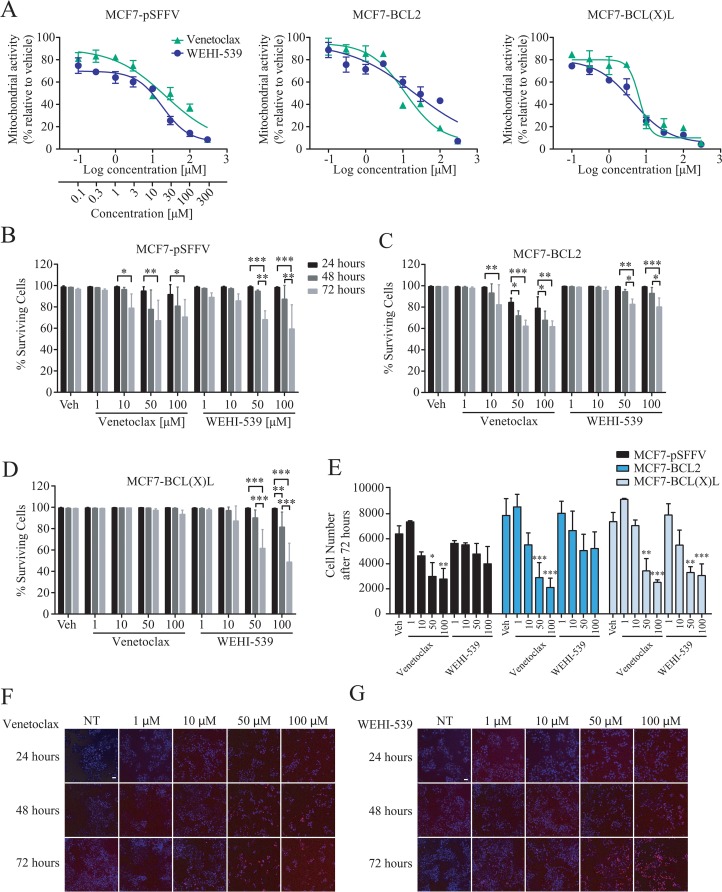
BCL2 inhibitors decrease mitochondrial function **(A)** Mitochondrial activity was measured with MTT assay after 72 hours exposure with increasing concentration of Venetoclax or WEHI-539 (from 0.1 to 300 μM). **(B, C and D)** Surviving cell counts after BCL2 or BCL(X)L inhibition in MCF7-pSFFV, MCF7-BCL2 and MCF7-BCL(X)L cells, respectively. Cells were treated with 1, 10, 50 and 100 μM Venetoclax or WEHI-539, separately, and stained with Hoechst/PI solution. After 24, 48 and 72 hours treatments, 96 well plates were imaged on a HCS microscope at 37°C and 5% CO_2_. An automated Cell Profiler pipeline was used to obtain percentage of live and dead cells. Surviving cells have Hoechst positive/PI negative nuclei shown in MC7-pSFFV, MCF7-BCL2 and MCF7-BCL(X)L, respectively. **(E)** The total number of cells after 72 hours was quantified with Cell Profiler and plotted to compare differences in the single clones. All data are expressed as mean ± SD from three independent experiments; in each experiment, treatments were performed in triplicate. Significance was assessed with a two-way ANOVA and Tukey post-test (^*^ indicates a p-value < 0.05, ^**^ indicates a p-value < 0.01 ^***^ indicates a p-value < 0.001). **(F and G)** Representative images of Hoechst/PI merged channels for Venetoclax and WEHI-539 treatments, respectively, in MCF7-pSFFV cell line. Scale bar: 50 μm.

We first determined the effect of selective BCL2 proteins inhibition on mitochondrial activity in our previously characterised MCF7 breast cancer cells overexpressing either BCL2 (MCF7-BCL2) or BCL(X)L (MCF7-BCL(X)L), or transfected with the corresponding empty vector (MCF7-pSFFV) [23–25]. Cells were treated with the selective BCL2 inhibitor Venetoclax or the BCL(X)L inhibitor WEHI-539 and evaluated using an MTT assay. MTT assays are routinely used as measures of cell growth and cell death, but primarily indicate mitochondrial NAD(P)H-dependent oxidoreductase enzyme activity. Cells were treated with selective inhibitors or vehicle (Supplementary Figure 1A) for up to 72 hours. As shown in Figure 1A, treatment with increasing concentrations of either selective inhibitor decreased NAD(P)H-dependent oxidoreductase enzyme activity in all of the cell lines investigated. Lower concentrations of the drugs (0.1 to 3 μM) decreased MTT absorbance to approximately 60% while higher concentrations (10 to 300 μM) decreased absorbance to 20% to 50% in all clones investigated (Figure 1A). In parallel, we employed a high content screening (HCS) platform to quantify cell survival using Hoechst/PI double staining following 24, 48 and 72 hours treatments with the BCL2 and BCL(X)L selective antagonists (1 - 100 μM, Figure 1F and 1G). MCF7-pSFFV treated for 72 hours with 10, 50 or 100 μM of Venetoclax showed a maximal 30% decrease in cell survival, indicating that at higher concentrations, BCL2 inhibition induced cell death. A similar decrease in cell survival was observed with 50 and 100 μM WEHI-539 (Figure 1B). In BCL2 overexpressing cells, higher concentration of Venetoclax (50 and 100 μM) were required to decrease cell survival (Figure 1C). Interestingly, treatment of MCF7-BCL2 cells with WEHI-539 at 50 and 100 μM also induced a 20 % decrease in cell survival (Figure 1C). In MCF7-BCL(X)L cells, only 50 and 100 μM of WEHI-539 decreased surviving cells after 72 hours, while no change was detected in the presence of Venetoclax (Figure 1D). Of note, these concentrations were 165 fold higher than those achieved for MTT reduction.

The high content imaging platform also allowed us to quantify the total number of cells present in the wells following BCL2 antagonist treatments. As shown in Figure 1E, 50 and 100 μM Venetoclax exposure for 72 hours significantly reduced cell numbers in all MCF7 clones, while WEHI-539 was effective only in BCL(X)L overexpressing cells. Collectively, these data suggest that the effects of Venetoclax and WEHI-539 on mitochondrial function as evaluated by the MTT assay were observed at concentrations 165 fold lower than those required for induction of cell death or inhibition of cell proliferation. Even at higher antagonist concentrations (100 μM) mitochondrial activity was decreased to 20-40%, while cell survival levels remained at 60-80%.

### BCL2 and BCL(X)L selective inhibition alter mitochondrial bioenergetics

**Figure 2:**
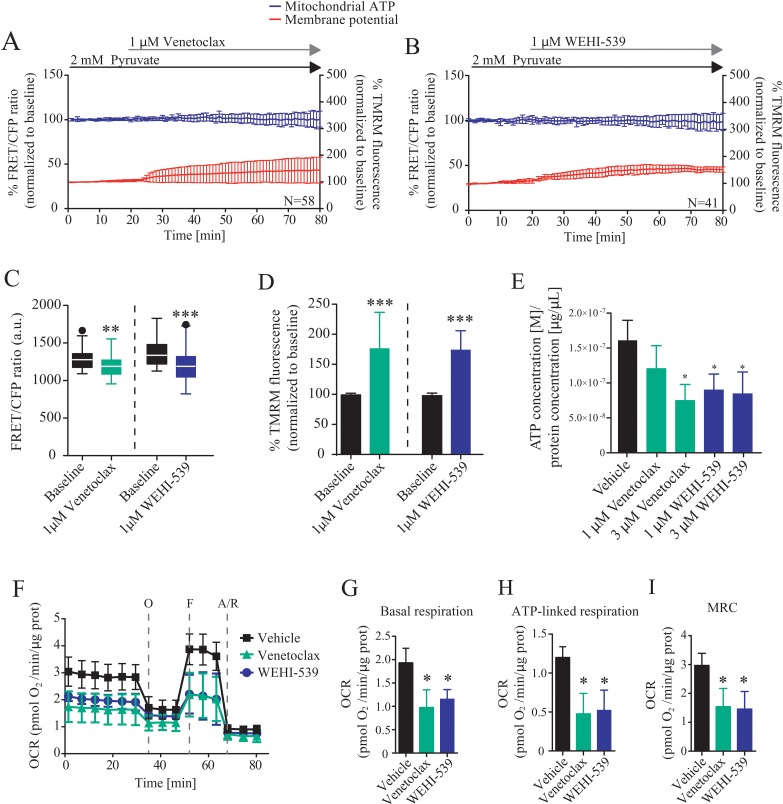
BCL2 and BCL(X)L selective inhibition decrease mitochondrial bioenergetics **(A and B)** Mitochondrial ATP and membrane potential traces in MCF7-pSFFV during Venetoclax or WEHI-539 treatments, respectively. FRET/CFP ratio kinetics and TMRM fluorescence were recorded simultaneously in MCF7 cells. Baseline was recorded for 20 minutes, after which Venetoclax or WEHI-539 (1 μM) were added to the medium and signals recorded for one hour. All data represent mean ± SD from n=5 independent experiments and both signals are normalised to the baseline levels. **(C)** The absolute FRET/CFP ratio was analysed by taking into account the minimal value reached by the probe in each cell after ABT199 or WEHI-539 treatment. Values were evaluated by one-way ANOVA with Tukey post-test for multiple comparison (^**^ indicates a p-value < 0.01 and ^***^ indicates a p-value < 0.001). **(D)** TMRM intensity values, normalised to the baseline levels, were analysed by taking into account the maximal value reached during ABT199 or WEHI-539 treatments and statistical analysis was performed as described in C. **(E)** Total ATP concentration normalized to proteins concentration after 1 and 3 μM treatments with Venetoclax or WEHI-539 for 72 hours in full RPMI medium. All data are expressed as mean ± SD from three independent experiments; in each experiment, treatments were performed in triplicate. Significance was assessed with a one-way ANOVA and Tukey post-test (^*^ indicates a p-value < 0.05). **(F)** OCR traces from Seahorse analyser experiments. MCF7-pSFFV cells were treated with vehicle, 1 μM Venetoclax or 1 μM WEHI-539, 1 hour prior the experiments. **(G, H, I)**. OCR values in Basal respiration, ATP-linked respiration and MRC, calculated from seahorse traces, respectively. All data are expressed as mean ± SD from three independent experiments; in each experiment, treatments were performed in triplicate. Significance was assessed with a one-way ANOVA and Tukey post-test (^*^ indicates a p-value < 0.05).

To further explore these findings, we directly investigated the ability of BCL2 and BCL(X)L selective inhibitors to alter mitochondrial ATP production and membrane potential by using a single cell imaging approach (Supplementary Figure 1B and 1C). MCF7-pSFFV cells were transfected with a mitochondrial ATeam expression vector, a FRET based sensor able to measure ATP production/consumption kinetics in living cells [26]. Cells were then placed in Krebs buffer (KB) in the presence of 2 mM pyruvate to supply mitochondrial respiration. Each inhibitor was separately added to the medium and mitochondrial ATP and membrane potential responses were recorded for one hour (Figure 2A and 2B). Both inhibitors induced a decrease in mitochondrial ATP levels, with BCL(X)L inhibition showing a greater effect than BCL2 inhibition (Figure 2C), as observed from absolute FRET/CFP ratio. Addition of vehicle did not produce any effect (data not shown). Interestingly, TMRM fluorescence intensity increased rapidly in MCF7-pSFFV when exposed to Venetoclax or WEHI-539 (Figure 2D). Again, addition of vehicle had no effect on mitochondrial membrane potential. We also confirmed the single cell studies in a population-based assay, employing a luciferase assay to measure overall ATP concentration. We found that treatment with Venetoclax (3 μM) or WEHI-539 (1 and 3 μM) also decreased the amount of ATP following 72 hours exposure at a population level (Figure 2E).

Next, we analysed whether or not the decrease in mitochondrial ATP levels were associated with a corresponding increase in mitochondrial NADH levels. A similar set of single cell imaging experiments as described in Figure 2A-2D was performed in MCF7 cells treated with WEHI-539. Analysis of NADH auto-fluorescence indicated a significant increase in the signal suggesting a decoupling in NADH-derived ATP production (Supplementary Figure 2A and 2B). Analysis of single cell slopes also revealed heterogeneous responses within the cell populations (Supplementary Figure 2C, 2D and 2E). We found that the majority of the cells treated with WEHI-539 showed increased NADH levels, but that the response was not uniform in all cells (Supplementary Figure 2E). A similar heterogeneity was observed when analysing mitochondrial ATP levels or membrane potential (Supplementary Figure 2C and 2D).

To confirm the results we also analysed the effect of the inhibitors on the oxygen consumption rates (OCR) using a Seahorse analyser. Cells were place in the assay medium with 2 mM pyruvate to mimic the conditions used for time-lapse experiments. We observed different OCR traces when cells where treated with 1 μM of Venetoclax or WEHI-539 (Figure 2F). Then, we analysed different parameters associated to the kinetics and observed reduced OCR in basal respiration (Figure 2G), ATP-linked respiration (Figure 2H) and maximal respiratory capacity (MRC, Figure 2I) when cells where treated with Venetoclax or WEHI-539, implying a loss of mitochondrial function. No differences were observed in the extracellular acidification rates (Supplementary Figure 2F).

### Venetoclax and WEHI-539 decrease mitochondrial network length

**Figure 3:**
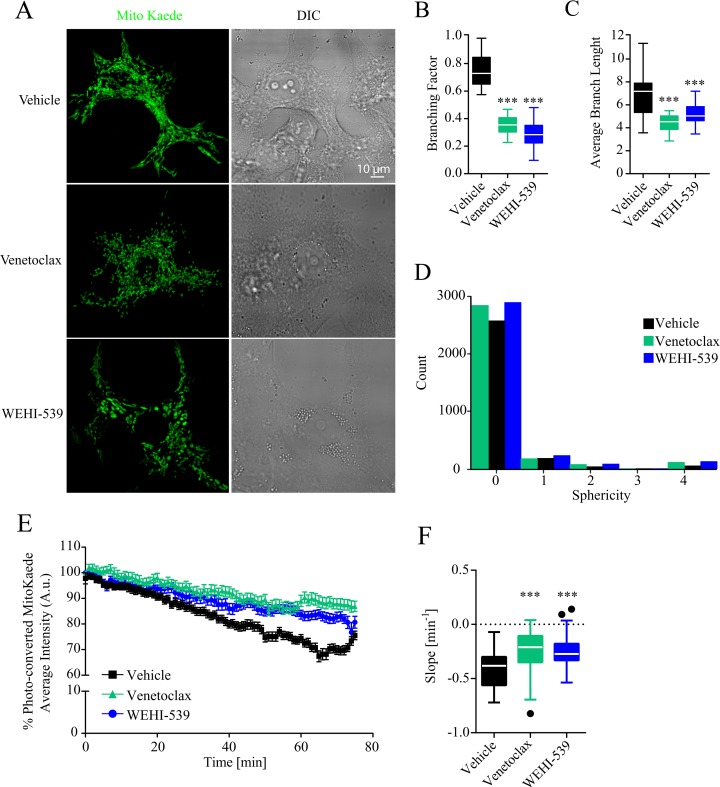
BCL2 and BCL(X)L selective inhibition decrease mitochondrial network length **(A)** Representative confocal images of mitoKaede expressing cells treated with vehicle, 1 μM Venetoclax or 1 μM WEHI-539 for 72 hours in full RPMI medium. **(B)** Branching factor calculated from z-stacks of Kaede expressing mitochondria. Images were deconvolved and skeletonized to obtain the branching factor. **(C)** Average branching length calculated from skeleton images of Kaede labelled mitochondria. **(D)** 3D sphericity of single mitochondrial objects. Images were processed with a white top hat filter and a 3D morphological filter was used to calculate the shape of the mitochondria. **(E)** Representative traces for time-lapse mitoKaede fluorescence intensity. Cells, in RPMI medium, were treated with vehicle, 1 μM Venetoclax or 1 μM WEHI-539, for 72 hours prior the experiment. The average signal values from each photo-converted area were normalised to the baseline. All data represent mean ± SD from 3 independent experiments and a total of 36 cells analysed. **(F)** Slope values from mitoKaede kinetic traces. The dynamics were assessed using a nonlinear fit – straight line function in GraphPad Prism. Significance was assessed with a one-way ANOVA and Tukey post-test (^***^ indicates a p-value < 0.001) for all the data in the figure panel.

Since ATP production and the general status of mitochondrial network are tightly interlinked, we also explored whether Venetoclax or WEHI-539 altered the mitochondrial network of MCF7 cells. To do so, we transfected cells with a mitochondrial targeted version of the fluorescent protein MitoKaede and treated the cells with 1 μM Venetoclax or WEHI-539 for 72 hours. We then measured the extent of mitochondrial network by performing z-stack imaging of live cells. As shown in Figure 3A, 3B and 3C both Venetoclax and WEHI-539 decreased the branching factor of the mitochondrial network and the average branch length, suggesting a less interconnected network and shorter mitochondria. We also analysed the sphericity of the single mitochondria and observed an increased count of higher values following both inhibitor treatments, suggesting an increase in rounder organelles (Figure 3D). Additionally, we also explored mitochondrial dynamic kinetics, using the photo-conversion properties of Kaede fluorescent protein. Cells were treated with either vehicle, 1 μM Venetoclax or WEHI-539 for 72 hours and fusion/fission rates measured with time-lapse confocal imaging for 75 minutes (Supplementary Figure 3). As shown in Figure 3E, the fluorescence signal from the photo-converted MitoKaede slowly decreased during the course of the experiment suggesting an active mitochondrial network. However, both Venetoclax and WEHI-539 treated cells showed decreased fusion/fission rates (Figure 3E). We analysed the slope of the fluorescence signal decay, and found increased slope values following the treatments with BCL2 and BCL(X)L inhibitor, suggesting a slower kinetic process (Figure 3F).

### Venetoclax and WEHI-539 synergize with 2DG and decrease the clonogenic potential of breast cancer cell lines

**Figure 4:**
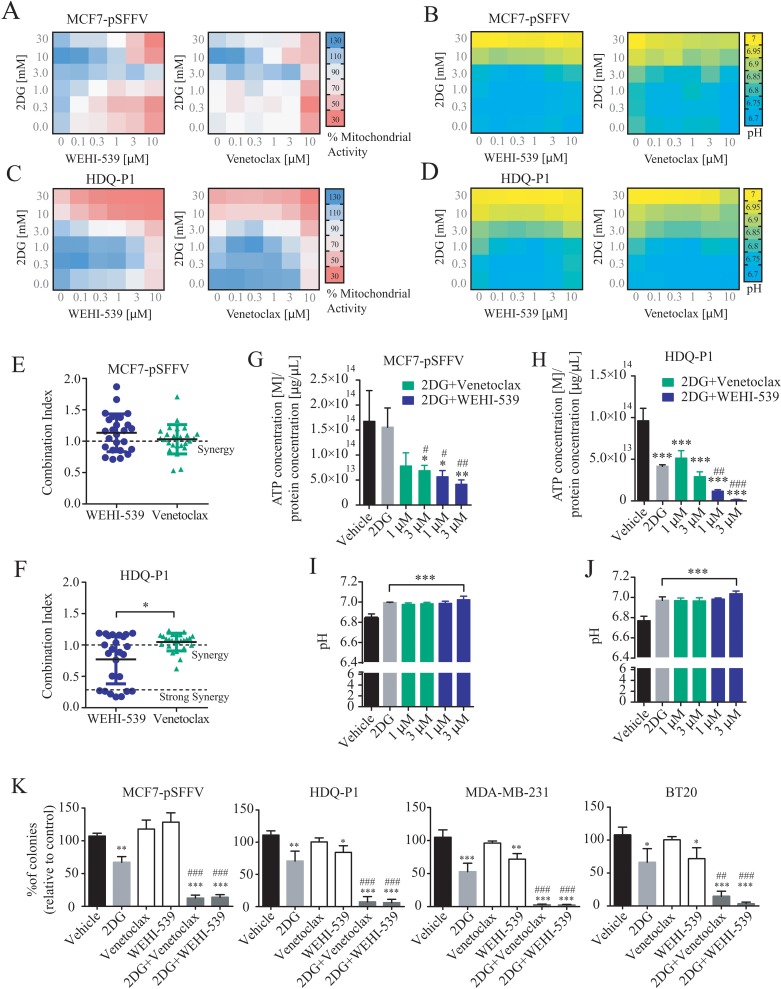
Combination treatment of 2DG with WEHI-539 or Venetoclax alters pH levels and mitochondrial activity and decrease clonogenic activity in breast cancer cell lines A 6×6 dose matrix assay was performed treating MCF7-pSFFV and HDQ-P1 cells with increasing concentration of 2DG in combination with increasing concentration of WEHI-539 or Venetoclax for 72 hours. Mitochondrial activity was measured by MTT while pH was measured by phenol red absorbance and represented as a heatmap. **(A and B)** Mitochondrial activity and pH values for WEHI-539 and Venetoclax, respectively, in MCF7 cells. **(C and D)** Mitochondrial activity and pH data for WEHI-539 and Venetoclax, respectively, in HDQ-P1 cells. All values represent mean from n = 3 independent experiments; for each experiment treatments were performed in duplicate. **(E and F)** CI values were calculated using Webb's fractional product method and analysed with one-way ANOVA with Tukey post-test to test significance in MCF7-pSFFV and HDQ-P1 cells, respectively (^*^ indicates a p-value < 0.05). A CI value lower than 1 means synergy while a CI lower than 0.3 is classified as strong synergy; CI values > 1 are considered as antagonistic. All experiments were performed in triplicate and results represent means ± SD. **(G and H)** Total ATP concentration normalized to proteins concentration after vehicle, 2DG alone or 2DG in combination with 1 and 3 μM Venetoclax or WEHI-539 for 72 hours in full RPMI medium, in MCF7 and HDQ-P1 cells, respectively. **(I and J)** pH measurement after same treatments in MCF7 and HDQ-P1 cells, respectively. **(K)** Clonogenic assay of MCF7-pSFFV cells treated with vehicle, 10 mM 2DG, 3 μM of each inhibitor and combination treatment of 10 mM 2DG with 3 μM of BCL2 or BCL(X)L selective inhibitor in MCF7-pSFFV, HDQ-P1, MDA-MB-231 and BT20 cells, respectively. After 72 hours treatment medium was changed and clonogenic capability assayed after 7 days in culture. Images were cropped using ImageJ and colonies were counted automatically with Open CFU software and the change in colony growth was normalized to vehicle-treated cells. Bars in the figures G, H, I, J, K represent means ± SD from three independent experiments. One-way ANOVA with Tukey post-test was used to assess significance (^*^ indicated a p-value < 0.05, ^**^ indicates a p-value < 0.01, ^***^ indicates a p-value < 0.001). Asterisk was used to indicate significance between treated conditions and vehicle control, while pound was used for significance between BCL2 inhibitors and 2DG.

The above experiments suggested that selective BCL2 or BCL(X)L inhibition impacted on mitochondrial ATP production, but was not sufficient to induce cell death (Figure 1B-1D) or reduce cell numbers (Figure 1E) at concentrations below 10 μM. We thus hypothesised that glycolysis supported cellular bioenergetics under conditions of BCL2 or BCL(X)L inhibition, and next investigated whether glycolysis inhibition with 2-deoxy-D-glucose (2DG) exerted synergistic effects with BCL2 or BCL(X)L inhibition. First, we performed MTT assays in a 6×6 dose matrix format to test for any synergistic activity between BCL2 inhibitors and 2DG in either ER+ MCF7-pSFFV or TNBC HDQ-P1 breast cancer cells. After a 72 hour treatment with increasing concentration of 2DG in combination with increasing concentration of each inhibitor, mitochondrial activity was evaluated and data analysed using the Webb fractional product method for evaluation of drug interactions [27]. We also measured the pH of the medium by phenol red absorbance as a read-out of lactate production during glycolysis. Increasing concentrations of 2DG slightly induced an increase in MTT absorbance in MCF7 (Figure 4A) while decreasing the MTT absorbance in HDQ-P1 cells (Figure 4C). Increasing the concentration of Venetoclax and WEHI-539 greatly decreased the MTT signal in MCF7 cells (Figure 4A) while only a subtle change was recorded in HDQ-P1 cells (Figure 4C). Of note, higher concentrations of combination treatments (10 and 30 mM 2DG in combination with 3 and 10 μM of each inhibitor) decreased mitochondrial activity to 50% in MCF7-pSFFV cells (Figure 4A). Similar results were obtained with HDQ-P1 cells (Figure 4C), in which combination treatments (10 and 30 mM 2DG in combination with 0.1 to 10 μM Venetoclax or WEHI-539) were more effective in decreasing mitochondrial activity than single agent treatments (Figure 4A and 4C).

As shown in Figure 4B and 4D, higher concentrations of 2DG (10 and 30 mM) also increased the medium pH to 7 in both MCF7-pSFFV and HDQ-P1 cells. Treatment with 10 and 30 mM 2DG in combination with Venetoclax or WEHI-539 (0.1 to 10 μM) maintained the pH at 7, indicating that cells started to take up lactate from the medium. Lower concentrations of 2DG (0.3 to 3 mM) in combination with BCL2 inhibitors (0.1 to 10 μM) did not change the pH compared to control conditions (Figure 4B and 4D). This suggested that combination treatments with higher concentrations of 2DG and BCL2 antagonists induced metabolic stress with an associated inhibition of glycolytic activity and mitochondrial respiration.

Synergy analysis carried out using the Webb method revealed that both WEHI-539 and Venetoclax possessed synergistic activity when used in combination with 2DG in both cell lines (Figure 4E, 4F and Supplementary Figure 4). The CI (combination index) value is an indicator of synergy (CI<1), additivity (CI=1) or antagonism (CI>1). In the case of WEHI-539, lower CI values, indicating synergy, were observed for concentration ranging from 1 to 10 μM in combination with 0.3 mM 2DG in MCF7-pSFFV cells (Supplementary Figure 4A). In addition, synergistic interactions were observed with 3 and 10 μM WEHI-539 when combined with 10 or 30 mM 2DG (Supplementary Figure 4A). Again, for MCF7-pSFFV cells, Venetoclax showed synergistic CI values at higher combination concentrations (10 μM Venetoclax with 0.3 mM 2DG or 3 μM Venetoclax with 10 or 30 mM 2DG, Supplementary Figure 4B). Overall no significant difference was observed between CI values of 2DG in combination with WEHI-539 or Venetoclax (Figure 4E) in MCF7-pSFFV cells. For HDQ-P1 cells, we found high synergistic CI values for 0.1 to 10 μM WEHI-539 in combination with 10 or 30 mM 2DG (Supplementary Figure 4C). On the other hand Venetoclax showed low CI values for 3 or 10 μM concentrations in combination with 10 or 30 mM 2DG (Supplementary Figure 4D). 2DG/WEHI-539 combinations were significantly lower when compared to 2DG/Venetoclax, in HDQ-P1 cells, suggesting a more potent synergistic activity (Figure 4F).

We then chose two optimal synergistic combinations (1 and 3 μM for each selective BCL2 inhibitor in combination with 10 mM 2DG) and measured the overall ATP concentration after combination treatments. We found that 2DG alone did not induced a significant change in ATP concentration in MCF7 cells (Figure 4G) while 2DG (10 mM)/Venetoclax (3 μM) combination treatment and 2DG (10mM)/WEHI-539 (1 and 3 μM) significantly decreased ATP concentration when compared to vehicle or 2DG treated cells (Figure 4G). Conversely, 2DG alone was effective on TNBC HDQ-P1; in this context only 2DG (10mM)/WEHI-539 (1 and 3 μM) significantly decreased ATP concentration (Figure 4H). All treatment induced a significant increase in pH when compared to vehicle treated cells (Figure 4I and 4J for MCF7 and HDQ-P1 cells, respectively).

One of the optimal synergistic concentration (3 μM for each selective BCL2 inhibitor in combination with 10 mM 2DG) was subsequently selected to perform clonogenic survival assays in MCF7 cells and three TNBC cell lines (HDQ-P1, MDA-MB-231 and BT20, Supplementary Figure 4E). Treatment of MCF7-pSFFV cells with 10 mM 2DG alone induced a 30 to 40% decrease in colony formation compared to vehicle treated cells (Figure 4K). On the other hand, treatment with either WEHI-539 or Venetoclax alone did not induce any change in colony formation (Figure 4K). However, both inhibitors, in combination with 2DG, showed a pronounced inhibition of colony formation, with a decrease to 10% of colonies when compared to vehicle or 2DG alone (Figure 4K). Similar results were observed in the TNBC cell lines where 2DG decreased the number of colonies to 60-70% and combination treatments to 5-10% when compared to either vehicle or 2DG treated cells. Of note, in these cells, WEHI-539 also significantly decreased colony formation when compared to vehicle treated cells.

### Combination treatments of Venetoclax or WEHI-539 with 2DG decrease surviving cells and block proliferation independently of glucose availability

**Figure 5:**
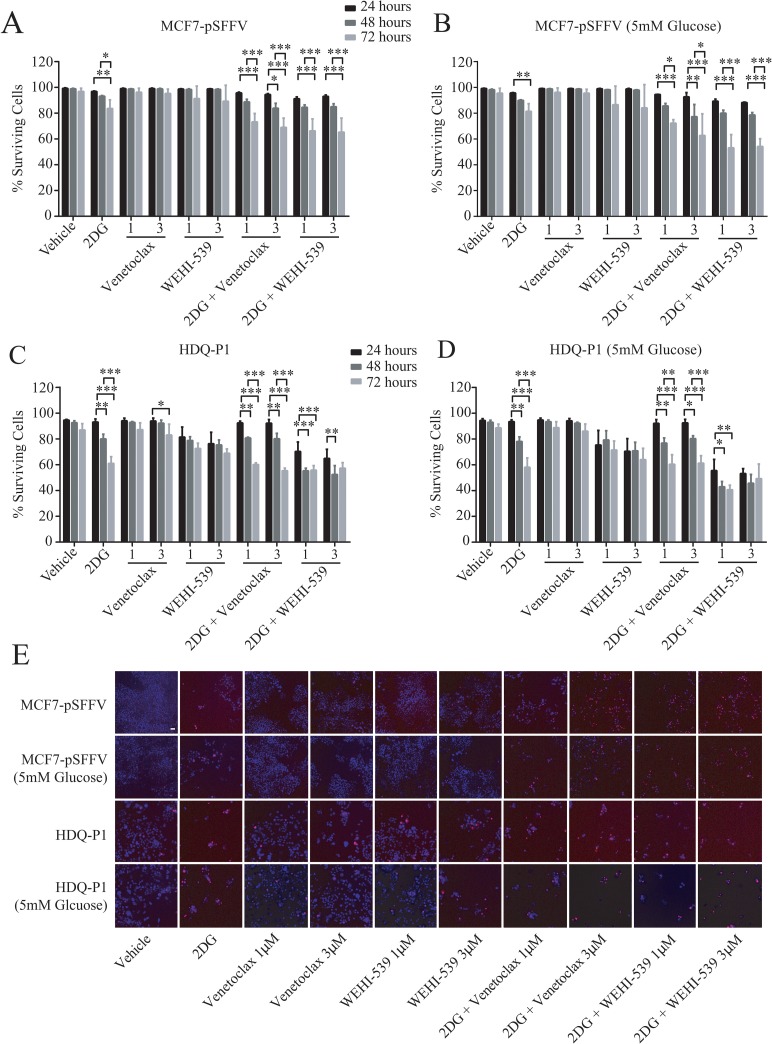
Combination treatment of 2DG with Venetoclax or WEHI-539 decrease cell survival in ER+ and TNBC cells Cells were treated with different concentration of Venetoclax or WEHI-539 alone or in combination with 2DG (10 mM or 5 mM, for full RPMI medium and 5 mM glucose medium, respectively) and stained with Hoechst/PI solution. At the selected time points (24, 48 and 72 hours) cells were imaged on a HCS microscope. An automated Cell Profiler pipeline was used to quantify alive and dead cells. **(A and B)** Surviving cell levels after treatments for MCF7-pSSFV in full glucose medium (RPMI 1640) or 5 mM glucose medium respectively. **(C and D)** Surviving cell levels after treatments for HDQ-P1 cells in full glucose medium (RPMI 1640) or 5 mM glucose medium, respectively. Bars represent mean ± SD from three independent experiments. Significance was assessed with a two-way ANOVA and Tukey post-test (^*^ indicates a p-value < 0.05, ^**^ indicates a p-value < 0.01 ^***^ indicates a p-value < 0.001). **(E)** Representative images of Hoechst/PI merged channels for all treatments in MCF7-pSFFV and HDQ-P1 cells, respectively. Scale bar: 50 μm.

We next investigated the effect of combination treatment on cell survival and proliferation in full RPMI medium (11 mM glucose) and low glucose medium (5 mM). MCF7-pSFFV and HDQ-P1 cells were treated with two concentrations (1 μM or 3 μM) of Venetoclax or WEHI-539 alone or in combination with 10 mM 2DG in normal medium or 5 mM 2DG in 5 mM glucose medium. Cells were stained with Hoechst/PI solution and the number of alive and dead cells was analysed with HCS microscopy after 24, 48 and 72 hours (Figure 5E). 2DG alone slightly decreased rates of surviving cells after 72 hours in MCF7-pSFFV cells in both condition (Figure 5A and 5B). The same treatment induced lower levels of surviving cells in HDQ-P1 cells after 48 (80%) and 72 hours (60%) in full glucose medium and 5 mM glucose medium (Figure 5C and 5D). Treatment with the inhibitors did not produce a significant effect on cell survival in MCF7-pSFFV. In HDQ-P1, a slight decrease in surviving cell levels was observed with WEHI-539 alone (Figure 5C and 5D). Combination treatment of 10 mM 2DG (in full medium) and 5 mM 2DG (in reduced glucose conditions) with both concentrations of Venetoclax or WEHI-539 significantly reduced surviving cells after 72 hours and also after 48 hours in the case of 3 μM Venetoclax (Figure 5A and 5B). In HDQ-P1 cells, all combination treatments were more effective; in fact, a decrease in surviving cells was observed after 48 and 72 hours (Figure 5C and 5D). Of note, combination treatment with both concentration of WEHI-539 significantly reduced surviving cell levels in full medium conditions and 5 mM glucose conditions after 48 and 72 hours. The treatments were effective also after 24 hours (Figure 5C and 5D). We also quantified the level of cell death using flow cytometry in conjunction with Annexin V/PI staining. Combination treatments of MCF7 and HDQ-P1 cells for 72 hours increased the fraction of apoptotic cells which was partially blocked by the pan-caspase inhibitor Z-VAD (Supplementary Figure 5).

### Combination treatments of Venetoclax or WEHI-539 with 2DG decrease cell survival and diameter of 3D breast cancer spheroids

**Figure 6:**
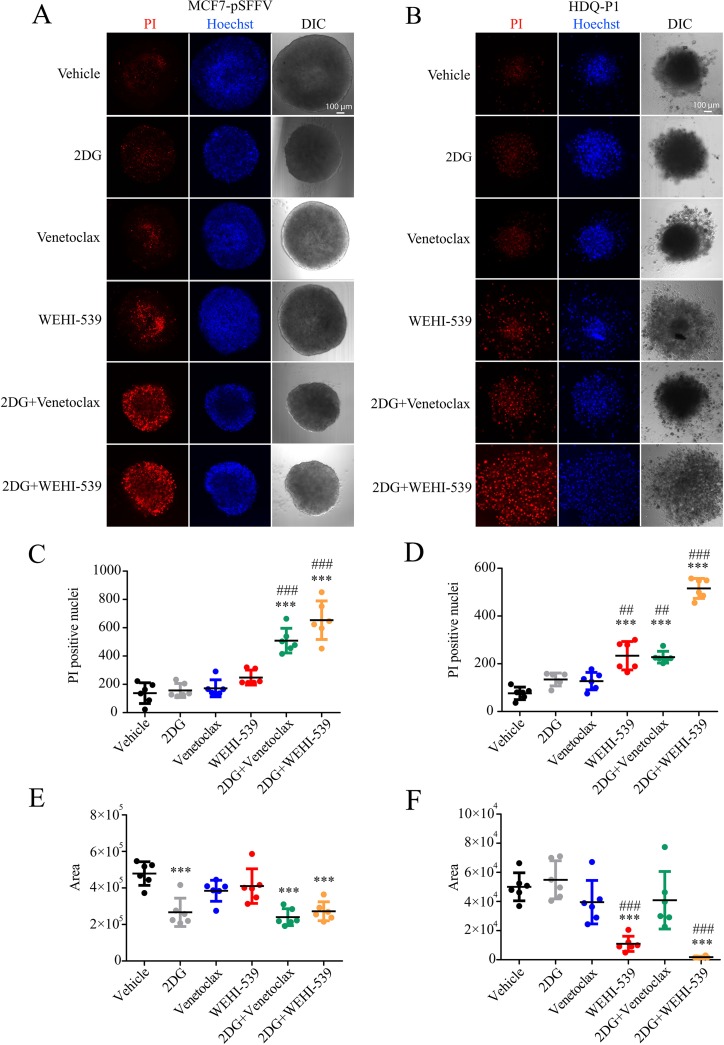
Combination treatments of 2DG with Venetoclax or WEHI-539 effectively increase the number of dead cells in breast cancer spheroids Breast cancer spheroids were stained with Hoechst\PI solution, treated with vehicle, 2DG alone (10 mM), Venetoclax or WEHI-539 alone (3 μM) and combination treatments for 72 hours. **(A and B)** Representative confocal images of Hoechst, PI and DIC signals for sections 60-80 μm inside each spheroid in MCF7-pSFFV and HDQ-P1 cells, respectively. **(C and D)** The number of PI positive cells was counted with ImageJ2 for MCF7-pSFFV and HDQ-P1 cells, respectively. **(E and F)** The area of spheroid section was quantified through the DIC channel using ImageJ2. Significance was assessed with a one-way ANOVA and Tukey post-test (^*^ indicates a p-value < 0.05, ^**^ indicates a p-value < 0.01 ^***^ indicates a p-value < 0.001). Bars represent mean ± SD from N=6 spheroids each treatment. Asterisk was used to indicate significance between treated conditions and vehicle control, while pound was used for significance between BCL2 inhibitors and 2DG.

Finally, in order to place the results in a more physiological setting, we performed the combination treatments on breast cancer spheroids. We used the Perfecta3D^®^ hanging drop plate to grow spheroids of 500 cells each, in full RPMI medium. Following one day incubation and visual confirmation of cell aggregation, we placed the cells in poly-hema coated 96 well plates containing each treatment. After 72 hours treatment we proceeded with confocal imaging of a section 60-80 μm inside the spheroid and quantified the area and the number of PI positive cells. We observed that both Venetoclax and WEHI-539 in combination with 2DG increased the number of PI positive cells when compared to vehicle and 2DG treated cells in both MCF7 and HDQ-P1 (Figure 6A, 6B, 6C, and 6D). 2DG and combination treatments also decreased the area of MCF7 spheroids when compared to vehicle treated cells (Figure 6A and 6E). Similar results were obtained for HDQ-P1 spheroids (Figure 6B and 6F). Of note, also WEHI-539 alone increased the number of PI positive cells when compared to vehicle and 2DG treated spheroids in HDQ-P1 (Figure 6B and 6D). However, 2DG in combination with WEHI-539 was more effective in inducing cell death in TNBC (Figure 6B and 6D). Interestingly both WEHI-539 alone and in combination with 2DG also induced a disaggregation of the spheroids (Figure 6B).

## DISCUSSION

In this work, we demonstrated that single agent treatment with the selective BCL2 inhibitors Venetoclax and WEHI-539 at therapeutically relevant concentrations decreases mitochondrial ATP production in breast cancer cells in the absence of cell death. Furthermore, we demonstrate that BCL2 inhibitors are synthetically lethal in breast cancer cells when combined with the glycolysis inhibitor 2DG, a finding that we also observed when cells were grown in 3D spheroids.

Initially, we determined the specificity of these inhibitors in MCF7 control cells and cells overexpressing BCL2 or BCL(X)L. Surprisingly, we found a reduction in MTT absorbance in MCF7 control cells and in BCL2 or BCL(X)L overexpressing cells with similar IC_50_s values. While we observed a profound decrease in the MTT signal 72 hours post antagonists treatment, little correlation was found to cell death data. We found that low (1 and 10 μM) or even higher concentrations (50 and 100 μM) of BCL2 or BCL(X)L inhibitors did not induce extensive cell death in MCF7 control cells as observed by Hoechst/PI staining. Furthermore, cell numbers did not vary for low concentration treatments, while a decrease was observed for higher concentrations. Similar results were observed in BCL2 or BCL(X)L overexpressing cells. As previously demonstrated, apoptosis is strongly inhibited in these BCL2 and BCL(X)L overexpressing cells [23]. Thus, it is plausible to hypothesise that the decrease in MTT absorbance recorded was mostly related to mitochondrial inhibition by BCL2 and BCL(X)L, rather than a measure of cell death or cell number. The fact that Venetoclax and WEHI-539 induced a similar decrease in MTT absorbance in control cells when compared to BCL2 and BCL(X)L overexpressing clones may be explained by a possible saturation of BCL2 or BCL(X)L at the binding sites important for the regulation of mitochondrial ATP production [28, 29], or by the targeting of other BCL2 family members by these inhibitors. In fact, Venetoclax still maintains a sensitivity to BCL(X)L (Ki = 48 nM) and BCLW (Ki = 245 nM) [30]. Likewise, WEHI-539 can bind at much lower affinity to other BCL2 proteins [31]. When surviving cell levels were analysed, Venetoclax exerted a higher effect on BCL2 overexpressing cell lines while no significant change was observed in BCL(X)L overexpressing clones. On the other hand, WEHI-539 was more potent in MCF7-BCL(X)L cells than it was on MCF7-BCL2 clone. These results are in line with a previous study that demonstrated a more potent effect of Venetoclax on cell death in a BCL2-dependent cell line compared to BCL(X)L when Annexin V and caspase 3/7 activity were evaluated after treatments [30]. Collectively, these data suggested pronounced effect of BCL2 and BCL(X)L inhibitors on cellular bioenergetics that were unrelated to apoptosis induction or effects on cell proliferation.

In order to confirm these results, we analysed the effect of both inhibitors on mitochondrial ATP, membrane potential and NADH kinetics. We found that Venetoclax and WEHI-539 treatments had a significant impact on mitochondrial bioenergetics, as demonstrated by a reduction in mitochondrial ATP levels when supplied with a mitochondrial substrate. We also noted significant hyperpolarisation of the mitochondrial membrane potential. The altered mitochondrial membrane potential observed could be related to the regulation of a proton leak by BCL(X)L and potentially BCL2 at the level of complex V. It has been previously shown that inhibition of BCL2 proteins with ABT737 led to a decrease in total ATP synthesis in neurons and increased the membrane leak conductance in sub-mitochondrial vesicles of the inner membrane [14]. Furthermore, it has been shown that ABT263 treatment and BCL2 inhibition induces OXPHOS impairment in primary acute myelogenous leukaemia (AML) and normal marrow CD34+ cells [32]. BCL2 proteins have been also found to regulate the channel activity of proteins such as VDAC [33, 34] and to form ion channels in lipid bilayers [35–37]. Moreover, it has been shown that BCL2 family members regulate the channel activity of adenine nucleotide translocator [38, 39] which exports ATP from the matrix and import ADP. This exchange activity of BCL2/BCL(X)L on adenine-nucleotide prevent mitochondrial hyperpolarization [40]. Hence, treatment with WEHI-539 or Venetoclax might inhibit the binding of BCL2 proteins with members of the permeability transition pore altering the properties of mitochondrial membranes and consequently their bioenergetics activity. In this context, work by Chen *et al.,* showed that treatment with ABT737 induces fluctuation in membrane potential, as BCL(X)L has a role in stabilising the potential by limiting total ion flux across the membranes [17]. Additionally, endogenous BCL2 in β-cells regulates ROS signalling and also reduces the redox sensitive proton leak in the mitochondria [41]. In line with the single cell imaging results and the aforementioned studies, we found altered OCR levels following the treatment with Venetoclax and WEHI-539. Most importantly, we observed decreased mitochondrial coupling efficiency (MRC) possibly due to defects in proton conductance or decreased substrate availability. We also observed decreased basal respiration and ATP-linked respiration, values affected by ATP demand and damage to OXPHOS [42].

Our experiments also highlighted that BCL2 protein inhibition induced heterogeneous responses in the cell population. The majority of MCF7 cells treated with WEHI-539 possessed increased NADH, increased TMRM with either decreased or stable mitochondrial ATP. Such heterogeneity can be explained by intrinsic differences in cellular BCL(X)L or BCL2 concentrations in breast cancer cell population, or by activation states of mitochondrial bioenergetics. Such heterogeneity in mitochondrial respiratory activity and ATPase activity has been previously demonstrated in cancer cells [43].

Mitochondrial bioenergetics and dynamics are tightly interconnected. Thus, it was crucial for this study to measure the mitochondrial network status after Venetoclax or WEHI-539 treatments. Previous literature demonstrated that BCL(X)L overexpression is able to increase fusion/fission and biomass in neurons [44], and a direct interaction between the fission regulator Drp1 and BCL(X)L was subsequently identified [45]. Strikingly, treatment of breast cancer cells with BCL2 and BCL(X)L selective inhibitors led to decreased mitochondrial network length. Mitochondrial membrane potential alterations induced by BCL2 inhibitors may also be linked to alterations in fusion/fission after Venetoclax/WEHI-539 treatments. It has been previously observed that Drp1-mediated mitochondrial fragmentation is reversed by an increase in mitochondrial membrane potential and that mitochondrial membrane potential is needed for the stimulation of fusion [46, 47]. The fusion mediator Opa1 requires a mitochondrial membrane potential for correct splicing by the intermembrane space protease Yme1L [48]. The decreased ATP production we observed is potentially linked to the change in mitochondrial network, since it has been shown that stressed mitochondrial networks process ATP at a lower rate [49]. In line with these studies, we also found decreased mitochondrial dynamics upon BCL-2 inhibition with Venetoclax and WEHI-539.

Because low concentration of the BCL2 inhibitors showed no effect on cell viability and/or proliferation but altered mitochondrial metabolism and network, we finally assessed the effects of combined glycolysis and BCL2 inhibition in MCF7 and TNBC cells. 2DG is a glucose analogue in which the 2-hydroxyl group has been replaced by hydrogen. Hexokinase II phosphorylates 2DG to form 2DG-P, which cannot be converted to fructose-6-phosphate by phosphohexose isomerase. The accumulation of 2DG-P leads to HKII inhibition [50]. Moreover, 2DG induces dissociation of HKII from mitochondria altering the link between glycolysis and mitochondrial respiration [51]. 2DG has been investigated in different clinical trials as a single agent. However, no significant improvements in patients were observed due to high adaptability of cancer cell to use different energy sources [52]. Thus, our working hypothesis was that dual inhibition of glycolysis and mitochondrial bioenergetics with 2DG and BCL2 inhibitors, respectively, could have a pronounced effect on cell viability and proliferation. First, we found synergism between both Venetoclax and WEHI-539 in combination with 2DG when mitochondrial activity was used as a read out. Our data suggested that synergistic interactions triggered a metabolic stress, as mitochondrial activity decreased and medium pH increased (as a result of lactate uptake). Additionally, synergistic combination also decreased the ATP concentration at a population level. Our results confirmed previous observations that TNBC cells are more glycolytic [10] as 2DG significantly reduced ATP concentration when compared to vehicle treated cells (Figure 4H). We also observed that MCF7 cells were more OXPHOS dependent [9] as no significant reduction was observed in the same conditions (Figure 4G). Combined treatments with 2DG and BCL2 inhibitors were also synthetically lethal and blocked completely the clonogenic potential of ER+ and TNBC cells. We also confirmed our results in a more physiological bioenergetics environment (5mM extracellular glucose). Additionally, we also found that treatment with a pan-caspase inhibitor inhibited the cell death induced by combination treatments. Recently, the ability of 2DG/ABT263 – ABT737 combinations to induce apoptosis in a xenograft model of prostate cancer was attributed to a disruption of the BAK-MCL1 and BAK-BCL(X)L complex by 2DG and ABT treatments, respectively [53], and hence start to undergo cell death. A second study proposed that 2DG treatment induced an AKT-independent, but glucose-dependent MCL1 degradation. Combination treatment of 2DG and ABT-199 led to JNK activation, with the subsequent phosphorylation and degradation of BCL(X)L [54]. Our data suggest that, in addition to effects of such combinations on BCL-2 protein levels and interactions, effects on cellular bioenergetics may significantly contribute to the reduced clonogenic potential of cells treated with such combinations, as cells with blocked cellular bioenergetics cannot proliferate and eventually undergo cell death.

Collectively, we here demonstrate that BCL2 inhibitors decrease mitochondrial bioenergetics and that the targeting of glycolysis, mitochondrial metabolism and cell death signalling by a combination of BCL-2 and glycolysis inhibitors could potentially benefit both ER+ and TNBC breast cancer subtypes. This could be of a potential interest for the development of new approaches that can rewire metabolism and cell death at the same time, to treat cancer therapy resistant patients.

## MATERIALS AND METHODS

### Materials and reagents

Fetal bovine serum, RPMI 1640 medium, Thiazolyl Blue Tetrazolium Bromide (MTT), dimethyl sulfoxide (DMSO), sodium pyruvate, D-glucose, 2-deoxy-D-glucose, Hoechst 33588 and propidium iodide (PI) came from Sigma-Aldrich (Dublin, Ireland). DMEM medium was purchased from Lonza (Analab Ltd, Lisburn, United Kindom). Tetramethylrhodamine methyl ester (TMRM) was from Invitrogen (Biosciences, Ireland). Venetoclax was purchased from Active Biochem (Maplewood, NJ, USA), WEHI-539 from ChemScene (South Brunswick, NJ, USA).

### Cell lines

MCF7-pSFFV, MCF7-BCL2 and MCF7-BCL(X)L were cultured in RPMI-1640 supplemented with 10% FBS, 1% L-Glutamine and 1% Penicilin/Streptomycin. DMEM supplemented with 10% FBS, 1% L-Glutamine and 1% Penicilin/Streptomycin was used for HDQ-P1. All cell lines were incubated at 37°C in humidified atmosphere with 5% of CO_2_. Cell lines were authenticated by STR typing from Source Bioscience (Nottingham, United Kindom).

### MTT assay

The MTT assay was used to determine mitochondrial activity following Venetoclax and WEHI-539 treatments. MCF7 clones were seeded at a density of 3×10^4^ cells for well on 96-well plates, kept at 5% CO_2_ and 37°C and treated with increasing concentration of Venetoclax or WEHI-539 (from 0.1 to 300 μM). After 72 h, 20 μL of 5 mg/mL MTT in 1X PBS was added to each well and the plate incubated at 37°C for 4 h. Consequently medium was removed and crystals were suspended in 100 μL DMSO. Absorbance at 570 nm was recorded on a Multiskan® EX plate reader (Thermo Scientific, Dublin, Ireland).

### ATP quantification

ATP was quantified using CellTiter-Glo^®^ luminescence assay (Promega). Cells were seeded at a density of 2×10^4^ in a 96 well plate and left to adhere over-night. After this incubation time cells were treated with 1 μM or 3 μM of Venetoclax or WEHI-539 alone or in combination with 10 mM 2DG for 72 hours. ATP was quantified following the manufacturer's protocol. Briefly, 100 μL of the assay buffer was added to each well (containing 100 μL of medium) and cells were incubated for 2 minutes at RT on a shaker. The plate was left at RT for another 10 minutes to stabilize the signal, and the content transferred to a black bottom 96 well plate and then loaded on a Clariostar reader (BMG Labtech) to measure luminescence with a settling time of 0.2 seconds and the top optic. Protein concentration was assessed with micro BCA (bicinchoninic acid) assay (Pierce) in order to account for differences in cell number. An excel template was used to subtract background and calculate ATP concentration from a standard curve, and moles of ATP normalised to protein concentration.

### High content screening microscopy

Cells were seeded in a Nunc Micro Well 96 well optical bottom plate (Thermo Scientific) at a density of 1.5×10^4^ cells per well. The day of the treatment cells were incubated in medium with 1 μg/mL Hoechst 33588 and 1 μg/mL PI. After 24, 48 and 72 h treatment, plates were imaged at 30 fields of view per well using a Cellomics Arrayscan VTI (Thermo Scientific) microscope set up with a temperature of 37°C and 5% of CO_2_ in humidified atmosphere. Images were taken at a resolution of 0.645μm/pixel using a 10x Plan-Apo objective lens (NA 0.45), a 120 W Hg arc illumination source with 12% ND filter (EXFO, Chandlers Ford, UK) and a monochrome CCD camera (Orca-AG, Hamamatsu Photonics, Hertfordshire, UK). The following filters sets were used: Hoechst excitation 387±11 nm, emission 447±30  nm; PI excitation 560±12 nm, emission 620±60 nm all using a HC-Quad band beam splitter with transition wavelength of 410, 504, 582, and 669 nm (Semrock, AHF, Germany). Images were analysed using a customised processing pipeline to identify nuclei with Hoechst staining (total cell number) and nuclei of dead cells (PI positive) using CellProfiler r2.2.0 [55].

### Measurement of cellular oxygen consumption

Cellular oxygen consumption rate (OCR) was measured using a Seahorse XF96 Extracellular Flux Analyzer (Seahorse Bioscience) [56].

MCF7-pSFFV cells were plated in XF96 V7 cell culture at 15×10^4^ cells/well and incubated for 48h in a 37°C, 5% CO_2_ incubator in RPMI medium. Cells were equilibrated with Seahorse XF DMEM Medium, pH 7.4 (Agilent), with the addition of 2 mM pyruvate, for 1 h immediately before extracellular flux assay. Cells were then treated with vehicle or 1 μM of Venetoclax or WEHI-539, for one hour prior the experiment, in the same medium. Drugs were prepared in the same medium in which the experiment was conducted and were injected from the reagent ports automatically to the wells at the times indicated. Mitochondrial function was determined through sequential addition of 6 μM oligomycin (O), 1 μM carbonyl cyanide-4-(trifluoromethoxy)phenylhydrazone (F), and 1 μM antimycin/1 μM rotenone (A/R). This allowed determination of basal oxygen consumption (the difference between the basal levels and the antimycin/rotenone treatment), oxygen consumption linked to ATP synthesis (the difference between the basal levels and the oligomycin treatment), non-ATP linked oxygen consumption (leak, the difference between the oligomycin treated levels and the antimycin/rotenone treatment), maximal respiratory capacity (MRC, the difference between the FCCP treated levels and the antimycin/rotenone treatment) [42, 56].

### Live cell time-lapse imaging of mitochondrial ATeam FRET probe, TMRM dye and NADH autofluorescence

Cells were seeded at a concentration of 2×10^3^ in sterile Willco dishes and let to adhere over-night. Then, the plasmid with the mitochondrial targeted ATeam construct [26] was transfected into MCF7-pSFFV cells with lipofectamine 2000 for 4 hours. On the day of the experiment, adherent cells were washed twice with krebs-hepes buffer (KB, 140 mM NaCl, 5.9 mM KCl, 1.2 mM MgCl_2_, 15 mM HEPES) and the medium replaced with 1 mL of KB containing 30 nM TMRM, 2 mM sodium pyruvate and 2.5 mM CaCl_2_. Mineral oil was added on top of the KB to prevent evaporation and the dishes transferred to a heated stage above a 63x/1.4 NA Plan-Apochromat oil immersion objective lens on an inverted confocal laser-scanning microscopes (LSM 710, Zeiss). Mitochondrial ATP kinetics measurements were carried out using lasers of 405, 488 and 561 nm for excitation of FRET/CFP, YFP and TMRM respectively with a pixel dwell time of 2.55 μs and images taken every minute. Detection ranges were set to 445-513 nm and 513-562 nm for CFP and FRET/YFP, while 562-710 nm was used for TMRM with pinholes set to 2 μm optical sectioning (FWHM). For NADH kinetics measurement MCF7 cells were prepared as before and the medium replaced with 1 mL of KB with 2 mM pyruvate and 2.5 mM CaCl2. Mineral oil was added on top of KB to prevent evaporation and the dishes transferred to a heated stage above a 40x/1.3 Numerical Aperture (NA) Plan-Neofluar of an inverted epifluorescence microscope (Axiovert 200M, Zeiss). NADH experiments were carried out using a HBO 100 mercury short-arc lamp for excitation with illumination wavelength of 340 nm for NADH excitation with an exposure time of 100 ms, and a binning of 4×4. Emission was collected at 450 nm and images taken every minute. All kinetics were measured for twenty minutes without treatment in order to obtain a baseline signal. Venetoclax or WEHI-539 (1 μM) treatments were then applied for 1 hour before ending the experiment. Images were processed using ImageJ2 (National Institutes of Health, Bethesda, MD, USA) and Metamorph 7.5 (Universal Imaging Co., Westchester, PA, USA). Time-lapse sequences were imported into ImageJ and background was first subtracted from each image. After creating combined images of the three fields of views for each channel sequence, a median filter with a radius of one pixel was applied. The combined images were then processed using Metamorph. Mitochondria within cells were segmented from background using the YFP time lapse images. The segmented mitochondrial areas were converted into a mask used to remove background values from any further analysis of the FRET/CFP stack. To this end the FRET image stack was first multiplied by the YFP-mask and divided by CFP image stack, and regions of interest were then selected for analysis. A custom made Metamorph journal was used to obtain the average intensity signal from all regions, and an excel macro was then applied to sort the values and to converted them to percentage normalised to the baseline. All experiments were performed at least three times independently of each other.

### Mitochondrial network and dynamics analysis

For mitochondrial network and morphology analysis, a mitochondrial-targeted version of Kaede fluorescent protein (FLX1.8MitoKaede) was employed. FLX1.8MitoKaede was a gift from Geoffrey Owens (Addgene plasmid # 28133). Cells were first seeded at a density of 2^*^10^3^ sterile Willco dishes. Cells were let to adhere and then transfected with 0.2 μg of the plasmid for 4 hours using lipofectamine 2000 in Optimum medium. After 24 hours cells were treated with vehicle (1 μl of DMSO) and 1 μM of Venetoclax or WEHI-539. After 72 hours incubation cells were placed on a heated stage of a LSM710 confocal microscope with a 63x/1.4 NA Plan-Apochromat oil immersion objective in humidified atmosphere at 37°C with 5% CO_2_. Lasers 488 nm for excitation of green fluorescent Kaede with a pixel dwell time of 1.58 μs was used. A spectral detection range of 485-552 nm was used for the green Kaede emission. Green fluorescent positive mitochondria were first selected and the position marked on the ZEN 2009 software (Zeiss). Z-stacks were created by selecting a series of optical slices covering the entire mitochondrial network with a step size of 0.3 μm at a resolution of 1024×1024 pixels using the 63 × 1.4 NA oil immersion objective. The green fluorescent channel was then deconvolved using Autoquant X (version 2.1.0, Media Cybernetics, UK). Images were further processed using ImageJ2. For branching analysis, a smooth 3D filter using a Gaussian method and a sigma value of 1.0 was first applied to deconvolved stacks. Following the selection of a threshold, the skeletonize function was used to create a skeleton image and then processed with the analyse-skeleton module using the lowest intensity voxel as prune cycle method. The branching factor was calculated by dividing the sum of the junctions to the sum of the end-point voxels. For the sphericity analysis, we employed the morphological 3D filter set in MorphoLibj module in ImageJ2. Briefly, we applied a white top hat filter with a ball element shape of 2 voxels for xyz directions. After this we used the particle analysis 3D module in MorphoLibJ to obtain the sphericity of mitochondrial entities from each image. Values were then sorted and analysed using R version 3.2.2.

For mitochondrial dynamics experiments, lasers 488 nm for excitation of green fluorescent Kaede and 561 nm for red fluorescent Kaede with a pixel dwell time of 1.58 μs were used. A spectral detection range of 485-552 nm detected the green Kaede while a longpass filter of 555-648 nm was used for photo-converted Kaede emission. Green fluorescent positive mitochondria were first selected and the position marked on the ZEN 2009 software (Zeiss). Following optimisation of the zoom and ROI repositioning, a small region was selected in the mitochondrial area of interest and photobleached using a 405 nm laser at 5% power. Subsequent Time-lapse images were recorded every minute for 75 minutes. For the image analysis, sequences were imported and the background subtracted. Stacks were created for the red channel (photo-converted Kaede) and a median filter with a radius of one pixel was applied. Stacks were opened with Metamorph and a threshold was applied to photo-converted Kaede stacks in order to cover the red fluorescent signal. The log option in Metamorph was employed to obtain average fluorescence intensity values. Average values were then processed through Microsoft Excel; a decrease in signal intensity of photo-converted Kaede represent fusion events as the red signal is diluted from surrounding non photo-converted mitochondria. Results were represented as percentage normalised to baseline values.

### Synergy calculations

MTT was employed to measure mitochondrial activity, while phenol red absorbance was used to obtain pH values of the nutrient medium covering the live cells. Cells were grown in a 96 well plate at a density of 1.5×10^4^ cells per well and treated with increasing concentration of 2DG (0.3 to 30 mM) in combination with increasing concentrations of Venetoclax or WEHI-549 (0.1 to 10 μM). After 72 h treatment MTT protocol was utilised as previously described. An excel template was used to calculate the mitochondrial activity after normalization to vehicle treated cells and Combination index, using Webb's fractional product method [27]. pH was recorded before the addition of MTT through the measurement of phenol red absorbance spectra using a ClarioStar reader (BMG, Germany). The wavelength range was 350-650 nm with a step width of 5 nm and a bidirectional mode was employed for the reading. The path length correction, considering the volume (200 μL) and the thickness of the plate was taken into account, using appropriate options on the ClarioStar reader. An Excel template was utilised to calculate the 560/440 nm ratio and the calibration for the ClarioStar reader formula $\log\;\left[\frac{\frac{560\text{ nm}}{440\text{ nm}}}{0.0002}\right]/1.18$ was employed to obtain pH values.

### Clonogenic assay

A thousand cells were seeded in a 6-well plate. After 72 h treatment with 3 μM of Venetoclax or WEHI-539 alone and in combination with 10 mM 2DG, fresh medium was added in each well and colonies were growth for 7 days. Cells were then fixed in 4% PFA for 10 minutes at room temperature and stained with crystal violet (0.5% in 1X PBS). Plates were scanned on a CanoScan LiDE 80 (Canon) at a resolution of 1200 ppi. Images were then cropped with ImageJ and analyzed with OpenCFU software [57].

### 3D culture

MCF7-pSFFV and HDQ-P1 cells were seeded at a concentration of 500 cell per well in Perfecta3D® hanging drop plate (3D Biomatrix) in RPMI medium with 5% methyl cellulose and stained with Hoechst\PI solution (1 μg\μl). After 24 hour incubation, spheroids were transferred to 96 well plates coated with 50 mg/ml poly-hema (in 95% ethanol/ 5% water) and containing RPMI with vehicle, 10 mM 2DG, 3 μM Venetoclax or WEHI-539 or combination treatments. After 72 hours treatment spheroids were imaged on a Zeiss LSM 710 confocal microscope as above using a 10x/0.3 NA C-Apochromat objective lens. Lasers 405 and 561 nm for excitation of Hoechst and PI, respectively, with a pixel dwell time of 1.58 μs were used. Spectral ranges of 563-735 and 415-503 nm detected PI and Hoechst emission, respectively. Images were taken at a resolution of 2048×2048 pixels. ImageJ2 was used to automatically count the number of PI positive cells.

### Statistical analysis

Data are given as means ± S.D. (standard deviation). Correlations were assessed using Spearman's rank correlation analysis. For statistical comparison, two-way analysis of variance (ANOVA) or one-way analysis followed by Tukey's *post hoc* test were employed. p-values <0.05 were considered to be statistically significant.

## SUPPLEMENTARY MATERIALS FIGURES



## Abbreviations

OXPHOS: Mitochondrial oxidative phosphorylation
TNBC: Triple negative breast cancer
ER: Estrogen receptor
BCL2: B-cell lymphoma
FRET: Foerster resonance energy transfer
ΔΨ_m_: Mitochondrial membrane potential
KB: Krebs buffer
2DG: 2-deoxy-D-glucose
TMRM: Tetramethylrhodamine methyl ester
OCR: Oxygen Consumption Rate
