# Supplementary material for: BCL2 and BCL(X)L selective inhibitors decrease mitochondrial ATP production in breast cancer cells and are synthetically lethal when combined with 2-deoxy-D-glucose
Source: Oncotarget. 2018 May 25;9(40):26046–63. doi: 10.18632/oncotarget.25433 (PMC5995245; doi:10.18632/oncotarget.25433)
Supplement: Supplementary file 1 [file oncotarget-09-26046-s001.pdf]

BCL2 and BCL(X)L selective inhibitors decrease mitochondrial ATP production in breast cancer cells and are synthetically lethal when combined with 2-deoxy-D-glucose

SUPPLEMENTARY MATERIALS

Flow cytometry

Cells were seeded on a 24 well plate at a density of $6*10^4$ cells for well and treated with 10 mM 2DG, 3 µM Venetoclax, 3 µM WEHI-539 and combination treatments. Same treatments were repeated in the presence of z-VAD. For this, cells were treated with 50 µM of z-VAD for 1 hour prior the addition of remaining treatments. After incubation time (72 hours) cells were collected by tripsinization and stained with Annexin V-FITC and PI (Biovision) for 20 minutes at room temperature in dark condition and analyzed using a CyFlow ML (Partec) flow cytometer and FloMax software. A minimum of 10,000 events were recorded for each sample.

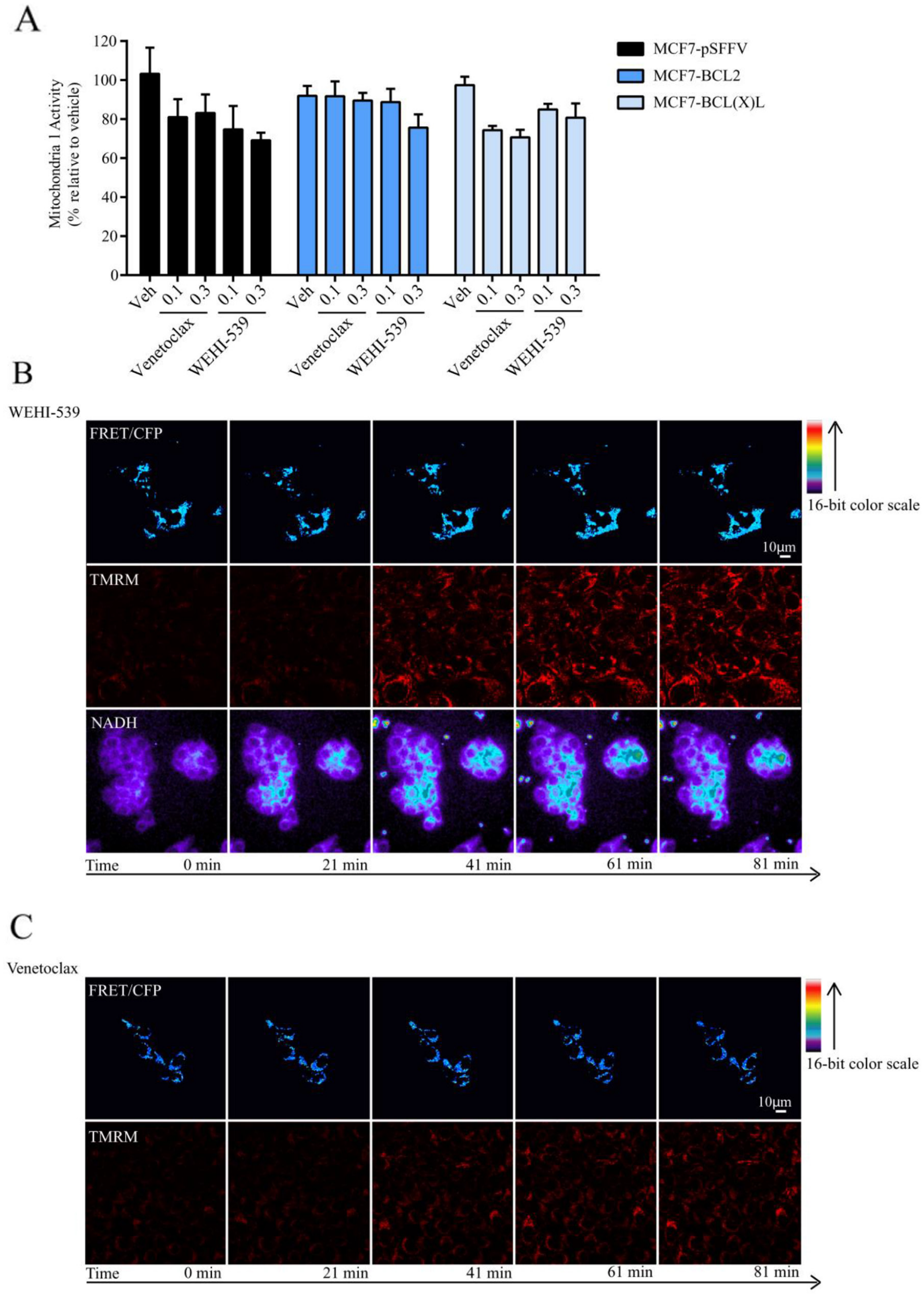

Supplementary Figure 1: Time-lapse imaging of mitochondrial ATP, mitochondrial membrane potential and mitochondrial NADH. (A) MTT values for vehicle treated cells and nanomolar concentrations of Venetoclax or WEHI-539. **(B and C)** Representative images for FRET/CFP ratio from mito ATeam FRET probe, TMRM and NADH autofluorescence for WEHI-539 and Venetoclax treatments, respectively.

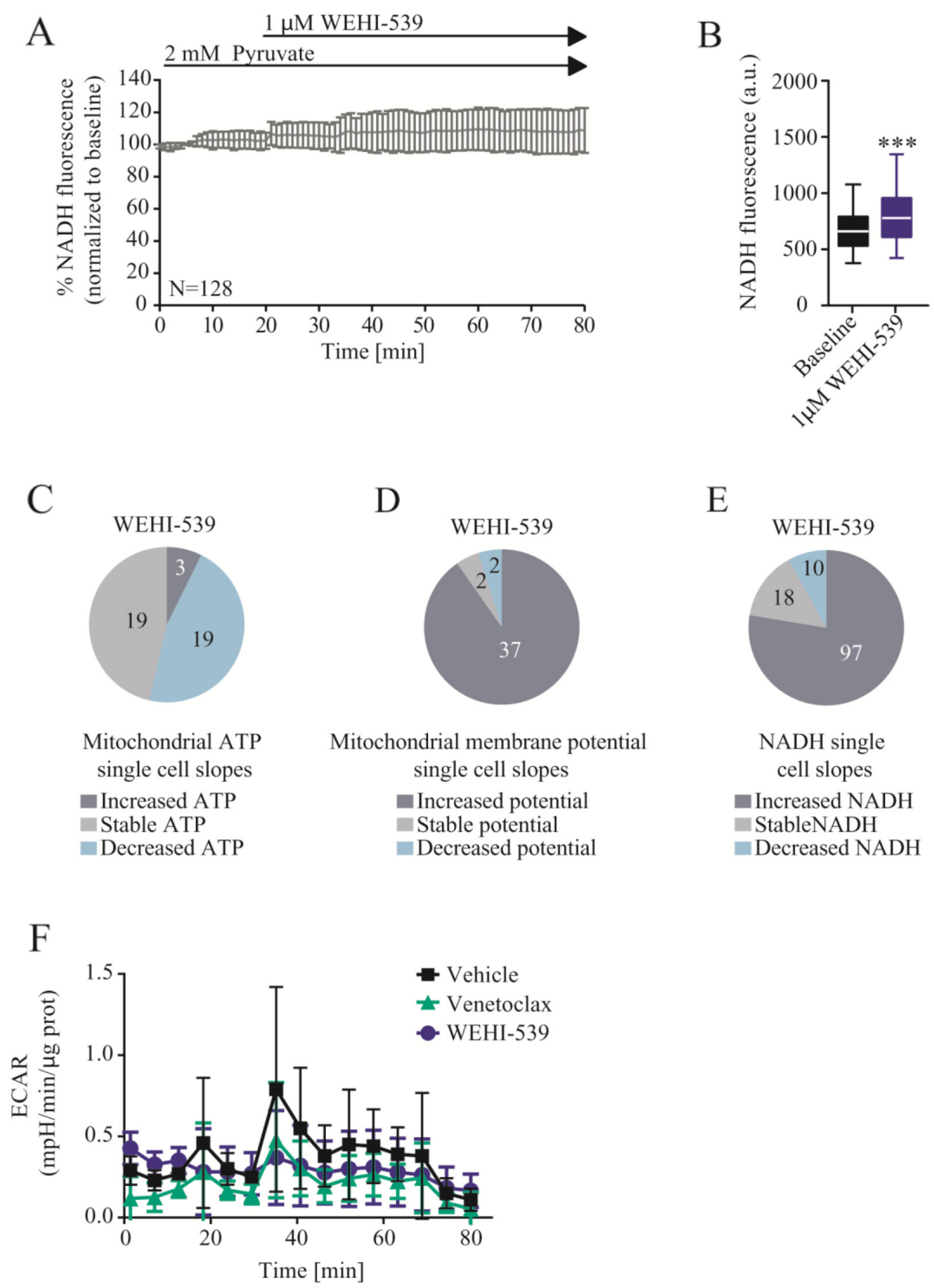

Supplementary Figure 2: NADH kinetics of NADH consumption during WEHI-539 treatment. (A) NADH auto-fluorescence traces in MCF7-pSFFV during WEHI-539 treatments. The experiment is similar to the one performed in figure 2. **(B)** The absolute NADH fluorescence was analysed by taking into account the maximal value reached by the probe in each cell after WEHI-539 treatment. Values were evaluated by one-way ANOVA with Tukey post-test for multiple comparison (*** indicates a p-value < 0.001). **(C, D and E)** The slopes for each cell lines was calculated using non-linear function in GraphPad and cells divided based on having increased, decreased or stable kinetics for mitochondrial ATP, mitochondrial membrane potential and mitochondrial NADH, respectively. **(F)** Extracellular acidification rates (ECAR) obtained from seahorse experiments in Figure 2.

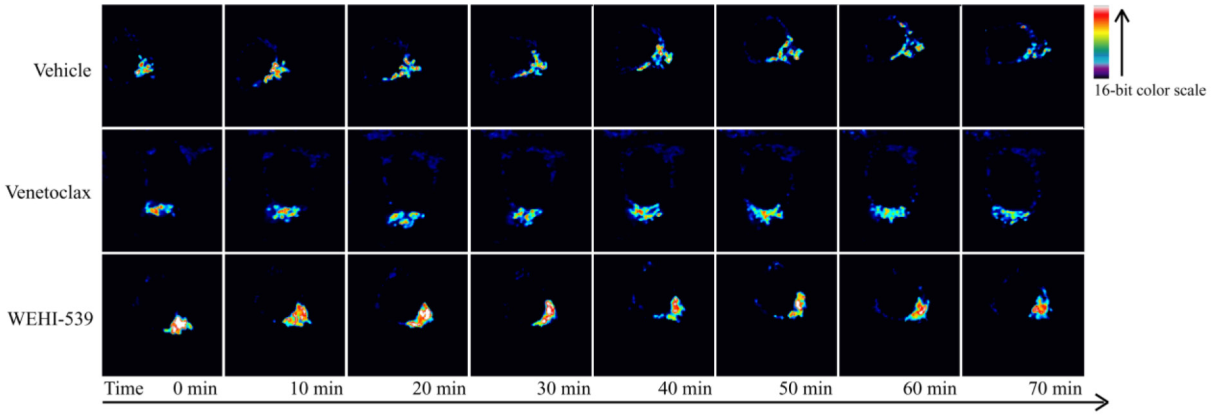

Supplementary Figure 3: Altered fusion/fission kinetics following BCL2 inhibitors treatment. Time-lapse images of photo-converted mitoKaede in MCF7 cells treated with vehicle, 1 μM Venetoclax or 1 μM WEHI-539.

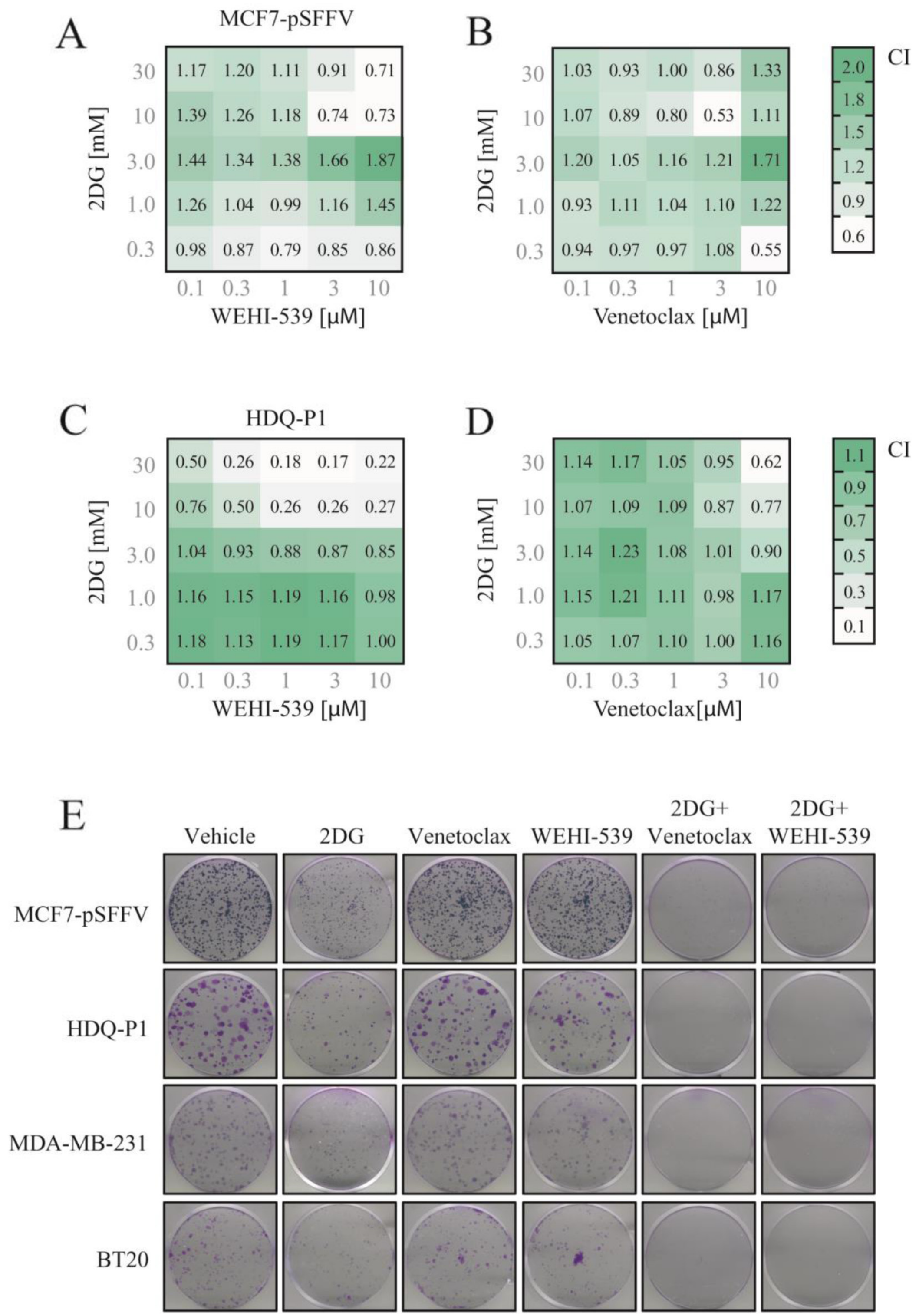

Supplementary Figure 4: Synergistic activity of 2DG in combination with Venetoclax or WEHI-539. (A and B) CI values were calculated using Webb's fractional product method from MTT data (Figure 4A) for 2DG/WEHI-539 and 2DG/Venetoclax combinations in MCF7-pSFFV cells, respectively. **(C and D)** CI values calculated from MTT data (Fig. 4C) for HDQ-Lucantoni et al. 3 P1 cells. **(E)** Representative images for clonogenic assay of vehicle, 2DG, Venetoclax and WEHI-539 alone and combination treatments.

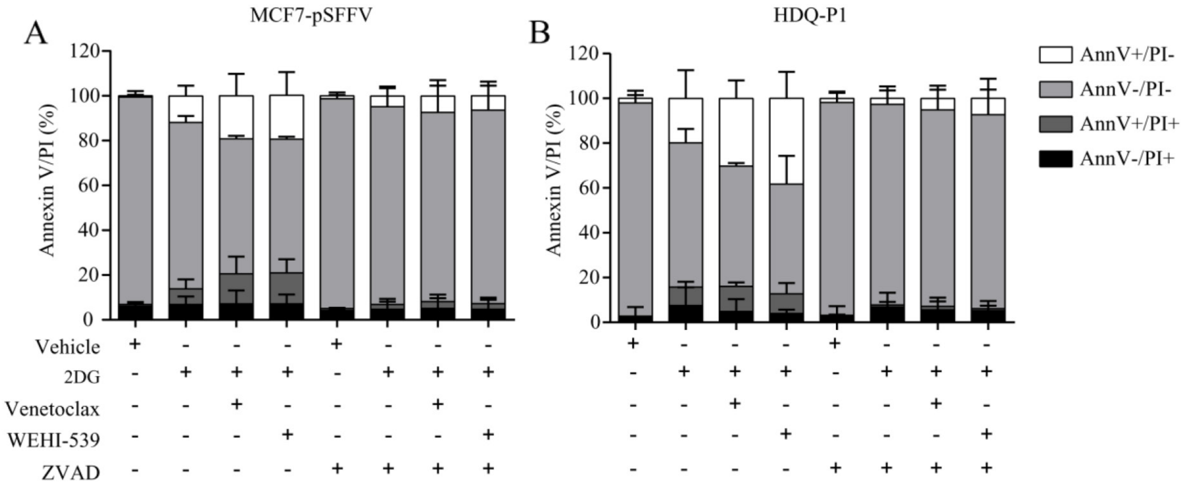

Supplementary Figure 5: Levels of cell death after treatment of 2DG in combination with Venetoclax or WEHI-539. (A) Percentages of surviving (AnnV-/PI-), early apoptotic (Ann V+/PI-), late apoptotic (AnnV+/PI+) and necrotic cells (AnnV-/PI+) after 2DG alone and combination treatments in the absence and presence of the pan-caspase inhibitor z-VAD in MCF7-pSFFV cells. **(B)** Same experiment repeated for HDQ-P1 cells.
